# Inhibitory effects of Chanling Gao on the proliferation and liver metastasis of transplanted colorectal cancer in nude mice

**DOI:** 10.1371/journal.pone.0201504

**Published:** 2019-02-21

**Authors:** Bing Yang, Chun-Shui Pan, Quan Li, Zhu Yang, Feng-Xi Long, Jing-Yu Fan, Chuan-She Wang, Jing-Yan Han, Dong-Xin Tang

**Affiliations:** 1 Guizhou University of Traditional Chinese Medicine, Guiyang, Guizhou, China; 2 Tasly Microcirculation Research Center, Peking University Health Science Center, Beijing, China; 3 Department of Integration of Chinese and Western Medicine, School of Basic Medical Sciences, Peking University, Beijing, China; 4 Department of Oncology, The First Affiliated Hospital of Guizhou University of Traditional Chinese Medicine, Guiyang, Guizhou, China; Columbia University, UNITED STATES

## Abstract

This study aimed to explore the efficacy and mechanism of Chanling Gao (CLG), a compound Chinese medicine, on colorectal cancer (CRC). A model of transplanted CRC was established in nude mice. The mice were treated 7 days after CRC transplantation with either Capecitabine or CLG for 3 weeks. On the 28th day after the operation, CRC growth and liver metastasis were assessed by morphology, the changes in the expression of HIF-1α (hypoxia inducible factor-1α), stromal cell–derived factor-1 alpha (SDF-1α), CXCR4 (C-X-C chemokine receptor type 4), PI3K, and Akt in the transplanted tumor and SDF-1α and CXCR4 in the liver were detected by Western blot and immunohistochemistry. The protein contents of vascular endothelial growth factor (VEGF), matrix metalloproteinase (MMP)-2, and collagen IV in the serum and transplanted tumor and SDF-1α and CXCR4 in liver tissues were detected by enzyme-linked immunosorbent assay. In the Capecitabine and high dose CLG groups, the growth and liver metastasis of CRC were significantly inhibited, the protein levels of HIF-1α, SDF-1α, CXCR4, MMP-2, VEGF, PI3K, Akt, P-PI3K and P-Akt in the transplanted tumor were lower, while the content of collagen IV in the transplanted tumor was higher, than in Model group. A high dose of CLG inhibited the growth of transplanted tumor and liver metastasis of CRC in nude mice, probably by inhibiting the HIF-1α/SDF-1α-CXCR4/PI3K-Akt signaling pathway reducing the synthesis and release of VEGF and degradation of collagen IV.

## Introduction

Colorectal cancer (CRC) is one of the most common malignant tumors, and its morbidity and mortality rank the third among all malignant tumors [1]. Distant metastasis is an important cause of poor prognosis in CRC patients, of which liver is the primary target organ for CRC hematogenous metastasis [2]. However, the liver metastases of most patients (80%–90%) cannot be treated with radical resection, and prevention of lever metastasis remains a challenge for oncologist [2].

Tumor metastasis is considered as an active, multistep, and organ-selective process[3], which is regulated by a wide range of chemokines [4]. Stromal cell–derived factor-1 (SDF-1) is a chemokine subfamily consisting of SDF-lα, SDF-1β, and SDF-lγ. SDF-1, which has receptor CXCR4 (C-X-C chemokine receptor type 4) on the cell surface of tumor cells. CXCR4 is a highly conservative G protein–coupled transmembrane receptor. Binding of SDF-1 to CXCR4 activates the downstream signaling pathway participating in regulation of cell proliferation, adhesion, and migration [5,6]. It has been proved that SDF-1/CXCR4 is highly expressed in CRC tissue, and the intensity of expression is closely related to tumor stage and lymph node and distant metastasis [7,8]. A study found that SDF-1 regulates the adhesion, chemotaxis, and cell morphology of human colon cancer cell line SW480 [9]. Thus development of medication targeting SDF-1/CXCR4 has attracted increasing attention.

Chanling Gao (CLG) was made by Professor Shangyi Liu, a Chinese national medical expert, with more than 50 years of clinical experience in Chinese medicine. It was made on the basis of Gegen San and refined using consisting of more than 20 traditional Chinese medicines, including Ge Hua (*Pueraria lobate*), Ge Gen (*Radix Puerariae*), Sha Ren (*Amomum villosum Lour*), Guan Zhong (*Dryopteris setosa*), Chan Pi (*Toad Skin*), and Wei Linɡ Xian (*Clematis chinensis*). CLG has been used in China in clinic since 2010 for nearly 10 thousand patients with tumors revealing favorite outcome. Previous studies showed that the Gegen San containing Ge Hua, Ge Gen, and Guan Zhong had a significant inhibitory effect on the cancer tissues of CRC nude mice, it could significantly promote the apoptosis of tumor cells [10], decrease the expression of ICAM-1, and regulate the number of regulatory T cells in the liver microenvironment in the case of CRC liver metastasis [11,12]. In addition, our toxicological study confirmed that CLG is safe for long-term clinical use [13,14]. However, the inhibitory effect and the possible underlying mechanism of CLG on CRC tumor formation and metastasis have not been investigated.

## Materials and methods

### Cells and animals

The LoVo, a human colon cancer cell line, was purchased from ATCC cell bank (Manassas, VA, USA). Five-week-old BALB/c male nude mice were provided by the laboratory animal facility of the Peking University Health Science Center (Beijing, Certification No.: SYXK2011-0039). The rats were reared at a temperature 23 ± 2°C and relative humidity 45 ± 5%, with 12-h light and 12-h dark alternation and allowed free access to food and water. All the experimental procedures were approved by the biomedical ethics committee of Peking University.

### Reagents and equipment

The F12K medium was purchased from Genview Scientific Inc. (Miami, FL, USA); PGL4.51 was purchased from Promega Corp. (Madison, WI, USA); Lipofectamine 2000 and Geneticin (G-418) were purchased from Life Technologies Corp. (Carlsbad, CA, USA); D-Luciferin (potassium salt) was obtained from Gold Biotechnology (St Louis, MO, USA); Medical OB glue was purchased from Guangzhou Baiyun Chemical Industry Co., Ltd (Guangzhou, China); CLG was provided by the First Affiliated Hospital of Guizhou University of Traditional Chinese Medicine (Guizhou, China); the Capecitabine tablets were purchased from Shanghai Roche Pharmaceuticals Ltd. (Shanghai, China); Antibodies against β-actin (1:2000; ab124964), CXCR4 (1:800; ab124824), SDF-1α (1:1000; ab25117), and HIF-1α(1:1000; ab16066) were purchased from Abcam Inc. (Cambridge, MA, USA). Antibodies against PI3K (1:1000; # 4257), Akt (1:1000; # 9372), P-PI3K (1:1000; # 4228), and P-Akt (1:2000; # 4060) were purchased from Cell Signaling Technology Inc. (Beverly, MA, USA). The enzyme-linked immunosorbent assay (ELISA) kits for SDF-1α, CXCR4, VEGF, MMP-2, and collagen IV were purchased from Andy Gene Co., Ltd. (Beijing, China). The SP kit was purchased from Beijing Zhongshan Jinqiao Biological Technology Co., Ltd. (Beijing, China); and the IVIS Spectrum imaging system was purchased from Xenogen Corp. (Alameda, CA, USA). All other chemicals were the products of AP.

### Transfection of LoVo stable cell lines with firefly fluorescent gene

The LoVo cells in the logarithmic growth phase were inoculated into a 24-well plate, and incubated for 24 h. When 85%–95% of the cells reached confluence, transfection was performed as described [15,16]. In brief, two mixtures were first prepared, one was the mixture of 50 μL of OPT-MEM medium and 1 μL of PGL4.51 plasmid, the other was the mixture of 2 μL of Lipofectamine 2000 and 50 μL of OPT-MEM medium. After standing at room temperature for 5 min, the two preparations were mixed, kept at room temperature for 25 min, and then added to the 24-well plate. After being incubated for 6 h, the solution in the wells was replaced by the F12K medium and then cultured in 5% CO_2_ at 37°C for 24 h. Afterwards, the F12K medium with 500 μg/mL of G-418 was added and screened for 2 weeks. The screened LoVo clone cells were transferred to a 96-well plate using a sterile filter paper. Once the concentration of G-418 decreased to 300 μg/mL, amplification screening was undertaken for 5 weeks, and positive monoclonal LoVo-luc cells were detected using the IVIS Spectrum imaging system.

### Establishment of *in situ* CRC in nude mice

The positive monoclonal LoVo-luc cells in the logarithmic growth phase were suspended in the F12K medium to a concentration of 2 × 10^6^ cells/mL, and 0.2mL of the suspension was inoculated subcutaneously into the right forelimb of BALB/c nude mice. When the tumor grew to about 1 cm^3^, the nude mice were sacrificed. The skin was sterilized, and the subcutaneous tumor was removed and immediately immersed in the F12K medium containing penicillin and streptomycin (100 U/mL each). The surrounding connective tissues were removed, and the tissues in which tumor grew vigorously were cut into 2×2×2 mm^3^ pieces for use. Pentobarbital sodium (40 mg/kg) was injected intraperitoneally, the skin was sterilized, and 1-cm incisions were made in the middle abdomen. The abdominal cavity was opened, and the cecum was removed. The serous membrane was punctured with a syringe needle at the end of cecum with abundant vascular supply. A tweezer was used to push inward to form a recess; then the prepared tumor was inserted into the recess. A sterile gauze was used to suck the surrounding liquid. An appropriate amount of medical OB glue was applied on the surface of the tumor, ensuring that it covered the surface of the tumor and spread to the cecum wall. The tumor was kept to stand still for 45 s. After the glue solidified, the cecum was brought into the abdominal cavity, and 5-0 surgical sutures were used to suture and close the incision. The whole process of operation and the waking of nude mice were undertaking on the thermostat plate at 37°C, which was beneficial to the survival of nude mice. After fully awaking, the nude mice were kept in a rat cage [17,18].

### Grouping and drug administration

In this study, the Chanling Gao low dose (CLGL) group was selected as the clinically equivalent conversion dose. Because of our preliminary experiments, the middle-dose group of CLG, which was twice the CLGL, was not significantly different from the low dose. Therefore, We chose the Chanling Gao high dose (CLGH) as 4 times the low dose. To clarify the efficacy of CLG we chose Capecitabine, an orally administered oral drug for colorectal cancer, as a positive control drug.

On the seventh day after the operation, the nude mice were randomly divided into four groups, 6 animals in each: Model group, the animals received no any treatment except for equivalent volume of normal saline by gavage; Capecitabine group, the nude mice were treated with Capecitabine for three courses, each course comprising consecutive 5 days of administration of Capecitabine (0.359 g/kg, 32.31 g/L) by gavage followed by 2 days of withdrawal [19]; CLGL group and CLGH group, the animals were given 0.2 mL of CLG solution by gavage at 1.52 g/kg(136.8 g/L) and 6.06 g/kg(545.4 g/L), respectively, once a day for 3 weeks.

During drug administration, the general status of the nude mice was observed daily, such as living conditions and diet activities. Starting on the first day of drug administration, the body weights of nude mice were measured every 2 days, and a body weight growth curve was plotted against time. At the end of the experiment, the nude mice were sacrificed by cervical dislocation.

### The *in vivo* imaging of the transplanted tumor of CRC in nude mice

The *in vivo* imaging of the transplanted tumor of CRC in nude mice was performed 7, 14, 21, and 28 days after operation. For this purpose, the animals were allowed to move freely for 10 min after they were injected intraperitoneally with fluorescein potassium substrate (150 mg/kg), and then kept in Matrx gas anesthesia system for anesthesia with isoflurane. The *in vivo* fluorescence images were acquired using the IVIS Spectrum imaging system, and the images were analyzed using the Living Image software (Caliper Life Sciences, Hopkinton, MA, USA). An equal size of the abdominal region was assigned as the region of interest (ROI), and the number of photons emitted per second (photon/s) in ROI was acquired for quantitative analysis[20].

### Assessment of tumor size and weight

The nude mice were sacrificed one day after the last drug administration. The tumor tissue was removed rapidly, and weighed on an electronic scale. The longest and shortest diameters of the tumor were measured using a vernier caliper. Tumor volume was calculated by the following formula: V (mm^3^) = *ab*^2^/2, wherein *a*, the longest diameter, *b*, the shortest diameter. Inhibition ratio of tumor weight = (1 – tumor weight in the experiment group/tumor weight in the model group) × 100%; Inhibition ratio of tumor volume = (1 – tumor volume in the experiment group/tumor volume in the model group) × 100%) [19].

### Evaluation of the liver metastasis of transplanted tumor in CRC nude mice

The complete liver was harvested one day after the last administration. The liver metastasis of the transplanted tumor was observed under a stereoscope, and the number of metastases of the transplanted tumor in the liver was counted. Some liver tissues were prepared for hematoxylin and eosin (HE) staining to determine the liver metastasis of the transplanted tumor.

### Western blot assay

Tumor tissue (100 mg) of nude mice in each group was adopted, cut into small pieces on ice, homogenized by ultrasound, and centrifuged at 4°C and 4000 g for 30 min. The supernatant was collected, protein concentration was determined by MicroBCA Protein Assay Kit (Pierce, Rockford, IL, USA), diluted with 5× loading buffer, and denatured. One hundred μg of protein from each sample was loaded and separated on sodium dodecyl sulfate–polyacrylamide gel, and transferred to polyvinylidene difluoride membrane. After blocked, the membrane was incubated with the primary antibodies against rat HIF-1α (1:1000), rabbit CXCR4 (1:800), rabbit SDF-1α (1:1000), rabbit PI3K/P-PI3K (1:1000), and rabbit Akt/P-Akt (1:1000) at 4°C over night. Following washing, corresponding secondary antibodies were applied for 1 h at room temperature. The gray value of protein bands was analyzed by Quantity One software (Bio-Rad, Hercules, CA, USA) [21].

### Elisa

The whole blood of the nude mice was collected at the end of the experiment, centrifuged at 1500 g for 15 min. The supernatant was harvested for assessment of the level of VEGF in serum by ELISA.

Transplanted tumor tissue (50 mg) in each group was obtained, cut into small pieces on ice, homogenized by ultrasound, and centrifuged at 4°C and 4000 g for 30 min. The supernatant was used for detecting the protein levels of VEGF, MMP-2, and collagen IV by ELISA.

Liver tissue (50 mg) of nude mice in each group was collected and processed as above to detect the protein levels of SDF-1α and CXCR4 in liver by ELISA [21].

### Immunohistochemistry

Paraffin sections (5 μm thick) of the transplanted tumor and liver tissues were prepared and processed for immunohistochemical staining using SP kit. The primary antibodies applied were HIF-1α (1:200), SDF-1α (1:300), and CXCR4 (1:700). Following incubation with corresponding secondary antibodies, the result was revealed by DAB. For quantification, five visual fields were sampled randomly in each section, and the number of both positive and negative cells was scored and expressed as the cell number per unit area. Four grades were classified according to the percent of positive cells: (1) Negative expression (<5%); (2) Weakly positive expression (5%–25%); (3) Positive expression (25%–75%); and (4) Strongly positive expression (75%–100%) [22].

### Statistical analysis

All data were expressed as mean ± standard error of the mean (SEM). GraphPad Prism 5 (GraphPad Software Inc, San Diego, CA, USA) and one-way analysis of variance were used for data statistics. Bonferroni correction was used for comparisons between the groups. *P* < 0.05 was considered significant.

## Results

### CLG inhibits the growth of the transplanted tumor in CRC nude mice

**Fig 1:**
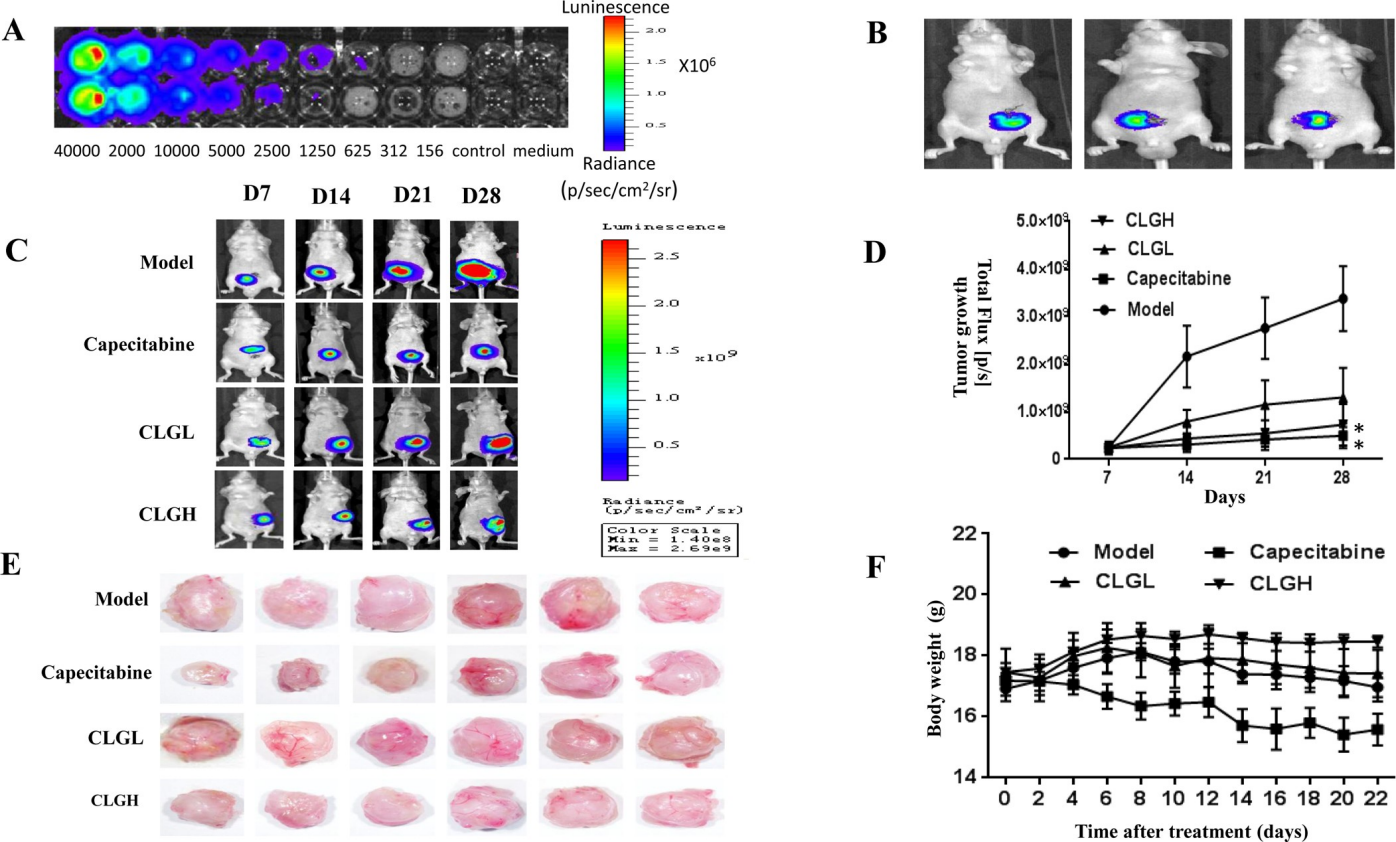
Inhibitory effect of CLG on the growth of CRC. **(A)** The *in vitro* luminescence of LoVo-luc. **(B)** The image on the seventh day after the establishment of the model in nude mice (*n* = 3). **(C)** The images in the IVIS system from different groups on the 7th, 14th, 21st, and 28th days after the operation. **(D)** The tumor growth of the nude mice in each group at corresponding time point. Data are mean ± SEM (*n* = 3), expressed by the change in total light flux. *P < 0.05.vs Model. **(E)** The images of the dissected transplanted tumors in each group observed under a stereoscope (*n* = 6). **(F)** Effect of CLG on the body weight of CRC nude mice (*n* = 6), the CLGH had a good maintenance effect on the body weight of CRC nude mice.

The results of luciferase activity assay showed that a stable cell line expressing luciferase (LoVo-luc cells) was established, and luminous intensity was proportional to cell density (Fig 1A). On the seventh day after the establishment of the model, *in vivo* imaging of the IVIS Spectrum showed a light spot in the nude mice (Fig 1B), indicating that LoVo-luc cells were successfully planted in the nude mice and began to grow. The light emitted increased with time in Model group (Model control group, given saline group) over the observation (Fig 1C), which was attenuated significantly in the Capecitabine and CLGH groups (*P* < 0.05) (Fig 1D). An attenuated emitted light was found in CLGL group as well compared with Model group, but no significance being observed. Moreover, the weight and volume of the transplanted tumor in the nude mice in the Capecitabine, CLGL, and CLGH groups decreased significantly compared with the Model group (*P* < 0.05) (Fig 1E and Table 1), further indicating the inhibitory effect of CLG on the growth of transplanted tumor in CRC nude mice.

**Table 1:** Inhibitory effect of CLG on the growth of the transplanted tumor in CRC nude mice (mean ± SEM, *n* = 6).

Group	Tumor weight (g)	Inhibition rate of Tumor weight	Tumor volume (mm^3^)	Inhibition rate of Tumor volume
Model	0.444±0.041	-	500.36±40.26	-
Capecitabine	0.16±0.028*	63.32%	171.95±35.12*	65.63%
CLGL	0.29±0.025*^#^	35.26%	318.87±38.08*^#^	36.27%
CLGH	0.24+0.011*	46.81%	280.82±25.34*	47.09%

CLGL: CLG low dose; CLGH: CLG high dose.

*P<0.05.vs Model

^*#*^*P<*0.05.vs Capecitabine.

### Effect of CLG on the body weight of CRC nude mice

During the period of treatment, the general situation, such as activities and mental state, of the nude mice in the CLG group was better than that in the Model and Capecitabine groups. The body weights of the nude mice in the Model group increased initially and then decreased gradually starting from day 8. On the other hand the body weights of the nude mice in the Capecitabine group decreased with time after treatment until day 14. The body weights of the nude mice in the CLGL group changed in a manner identical to that in Model group. Interestingly, the body weight in the CLGH group increased initially after treatment and then remained nearly constant (Fig 1F).

### CLG prevents the liver metastasis of the transplanted tumor

**Fig 2:**
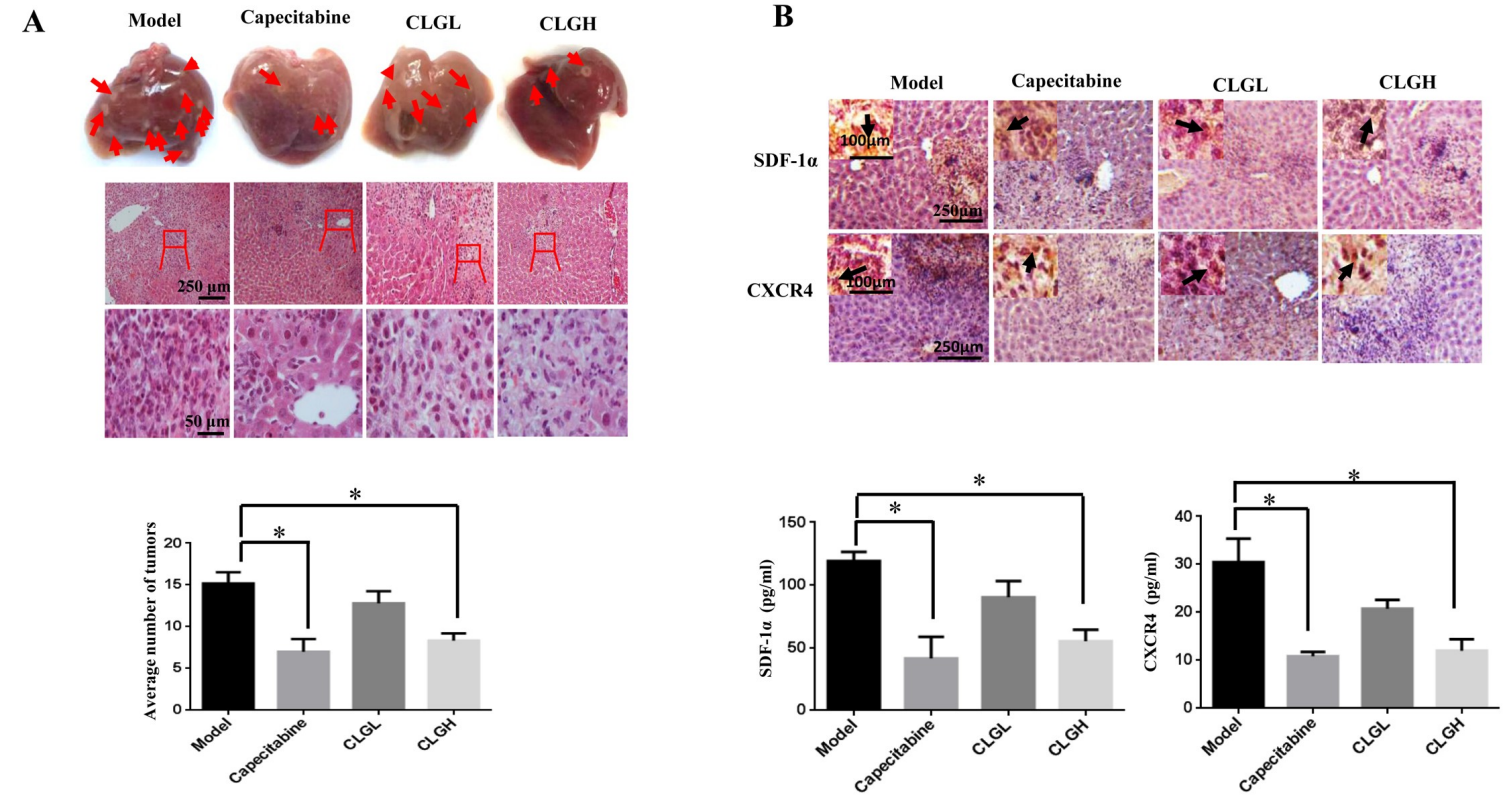
Effect of CLG on the liver metastasis of the transplanted tumor. **(A)** The metastasis of the transplanted tumor in the liver; the arrow points to the site of metastasis. The HE-stained sections of the liver tissues from different groups. The image within the rect in each low magnification picture was enlarged and displayed below. The number of metastases of the transplanted tumor in the liver from different groups. **(B)** Inhibitory effect of CLG on SDF-1 and CXCR4 in the livers of CRC nude mice. Immunohistochemistry of SDF-1α and CXCR4 in liver tissues in different groups. The protein level of SDF-1α and CXCR4 in the liver of nude mice tested by ELISA. Data are mean ± SEM (*n* = 6). *P<0.05 vs Model.

The liver metastasis of the transplanted tumor was assessed by stereoscope observation and HE staining at the end of the experiment (Fig 2A). The results showed that the liver metastasis of the transplanted tumor developed in all six nude mice in the Model group, three nude mice in the Capecitabine group and CLGH group respectively, and five nude mice in the CLGL group. However, the number of metastases in the liver of the Capecitabine and CLGH groups decreased significantly compared with that in the Model group (Fig 2A).

### CLG inhibits the expression of SDF-1α and CXCR4 in the liver

The expression of SDF-1α and CXCR4 in liver metastasis of CRC nude mice was assessed first by immunohistochemistry, revealing a lower expression of both proteins in the Capecitabine and CLGH groups than in Model group. This result was verified by ELISA (Fig 2B) (*P* < 0.05).

### CLG inhibits the expression of HIF-1α, SDF-1α, CXCR4, PI3K, and Akt in the transplanted tumor and the activation of PI3K and Akt

**Fig 3:**
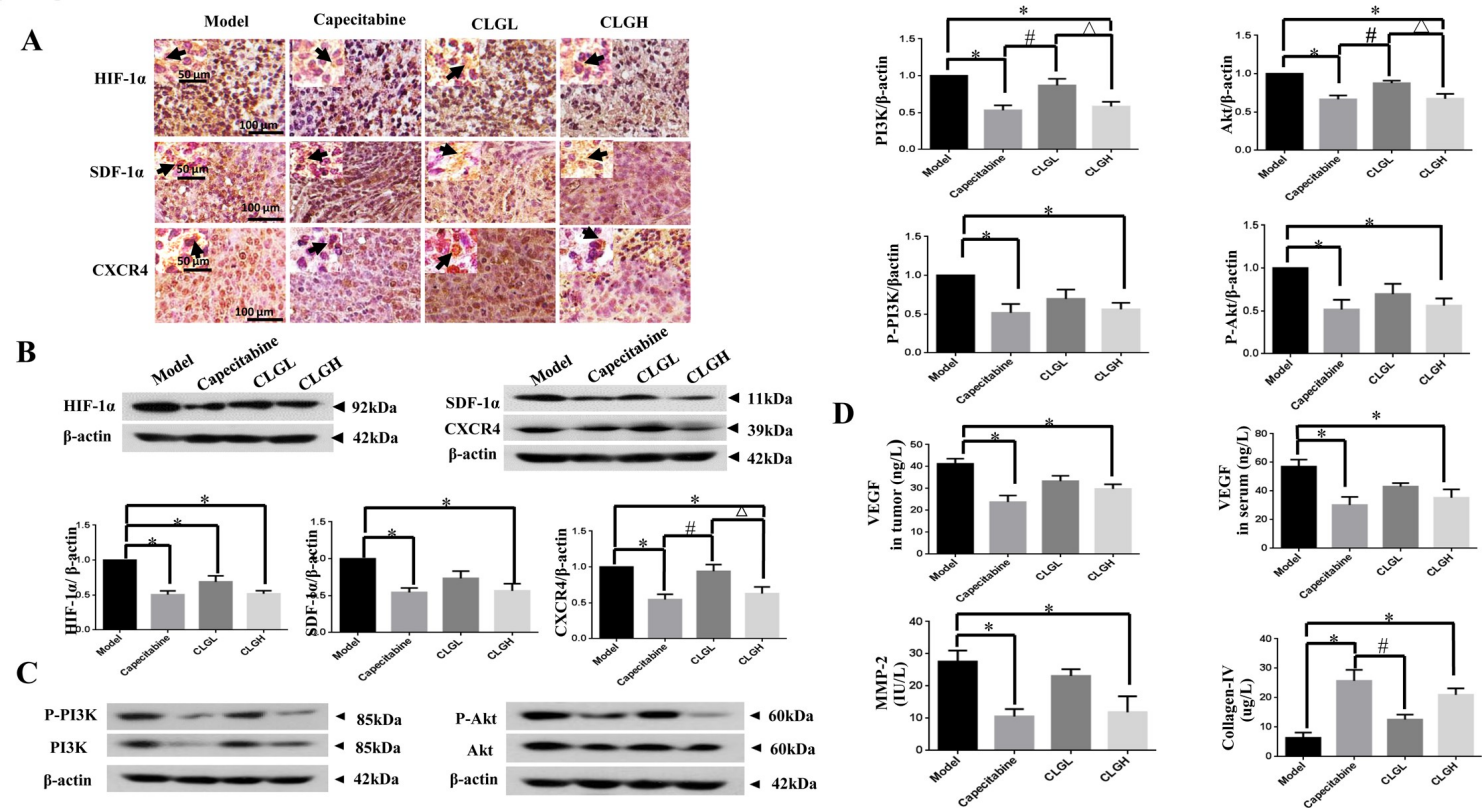
The regulating effect of CLG on related proteins. **(A)** Inhibitory effect of CLG on the expression of HIF-1α, SDF-1α, and CXCR4 in the transplanted tumor by Immunohistochemistry. **(B)** Inhibitory effect of CLG on the expression of HIF-1α, SDF-1α, and CXCR4 in the transplanted tumor tested by Western blot. A semi quantification of the protein levels of HIF-1α (92 kDa), SDF-1α (11 kDa), and CXCR4 (39 kDa) in Model, Capecitabine (32.31 g/L), CLGL (136.8 g/L) and CLGH (545.4 g/L) groups. Data are mean ± SEM (*n* = 6). *P < 0.05 vs Model, ^#^P < 0.05 vs Capecitabine, ^*△*^*P* < 0.05 vs CLGL. **(C)** Inhibitory effect of CLG on PI3K, Akt, and their phosphorylation in the transplanted tumor. The semi quantification of PI3K (85 kDa), Akt (60 kDa), P-PI3K (85 kDa) and P-Akt (60 kDa) protein level in Model, Capecitabine (32.31 g/L), CLGL (136.8 g/L) and CLGH (545.4 g/L) groups. Data are mean ± SEM (*n* = 6). *P < 0.05 vs Model, ^*#*^*P* < 0.05 vs Capecitabine, ^*△*^*P* < 0.05 vs CLGL. **(D)** Effects of CLG on the protein levels of VEGF, MMP-2, and collagen IV in serum and/or the transplanted tumor tested by ELISA. Data are mean ± SEM (*n* = 6). *P<0.05 vs Model, ^*#*^*P*<0.05 vs Capecitabine. All Western blot experiments were repeated three times.

The results of assessment of HIF-1α, SDF-1α, and CXCR4 by immunohistochemistry are displayed in Fig 3A, showing that the expression of the three proteins was strongly positive in the Model group, weakly positive in the Capecitabine group, positive in the CLGL group, and weakly positive in the CLGH group. This result was confirmed by Western blot. As shown in Fig 3B, compared with Model group, the expression of these proteins in the Capecitabine, CLGL, and CLGH groups significantly decreased. In this regard, Capecitabine and high dose of CLG exhibited a comparable efficacy.

The expression of PI3K, P-PI3K, Akt and P-Akt in the transplanted tumor from different groups was assessed by Western blot. As illustrated in Fig 3C, the expression of these proteins in the Capecitabine and CLGH groups significantly decreased compared with Model group. Again, Capecitabine and high dose of CLG exhibited a comparable inhibitory effect on both the expression and activation of PI3K and Akt. Low dose of CLG showed a trend to inhibit the PI3K / Akt signaling as well but without significance.

### CLG decreases the protein levels of VEGF and MMP-2 but increases the collagen IV

The levels of VEGF, MMP-2 and collagen IV in serum and/or the transplanted tumor were assessed by ELISA for different groups, and the results are shown in Fig 3D. Compared with Model group, the VEGF levels of both the transplanted tumor and serum decreased significantly in the Capecitabine and CLGH groups (*P* < 0.05). The MMP-2 protein contents of the transplanted tumor varied among groups in a similar fashion. In contrast, the collagen IV contents of the transplanted tumor in the Capecitabine and CLGH groups significantly increased as compared with Model group (*P* < 0.05).

## Discussion

The present study showed that CLG could inhibit the growth and liver metastasis of the transplanted CRC in nude mice, suggesting the potential of CLG as an adjuvant management to deal with CRC. Mechanistic studies revealed that CLG depressed the levels of SDF-1α and CXCR4, as well as HIF-1α, MMP-2, VEGF, PI3K, Akt, P-PI3K and P-Akt, while elevated the level of collagen IV, in the transplanted tumor of the nude mice, pointing to the involvement of HIF-1α/SDF-1α-CXCR4/PI3K-Akt signaling pathway in the action of CLG, which result in the reduction of synthesis and release of VEGF, and decreased degradation of collagen IV by MMP-2.

The invasion and metastasis are the major causes of death in patients with CRC, which implicate complicated processes mediated by multiple mechanisms [23]. Accumulating evidence shows that SDF-1 and its receptor CXCR4 are closely related to the invasion and metastasis of CRC [5–9]. A study reported that the level of CXCR4 in colon cancer is significantly higher than that in the normal tissues, and increases with the progression of tumor from the early (stages 0–II) to the late stages (III–IV stage) [24]. As a member of chemokine subfamily, SDF-1 binding with its receptor CXCR4 triggers CRC cells migration, promoting tumor metastasis. Thus, a regime able to inhibit the expression of SDF-1 and CXCR4 is expected to depress the CRC metastasis. In the present study, the results of Western blot and immunohistochemistry both showed that CLG significantly decreased SDF-1α and CXCR4 of the transplanted tumor, which, at least partly, underlies the protective effect of CLG on CRC metastasis.

The rapid growth of tumor results in the lack of blood supply, leading to hypoxia in tumor tissue and secretion of HIF-1. HIF-1 is a heterodimer consisting of two subunits HIF-1α and HIF-1β, which regulates the transcription of dozens of target genes that are involve in anaerobic glucose metabolism, oxygen delivery, angiogenesis, and angiectasis [25]. HIF-1 participates in regulation of tumor progress in multiple ways. HIF-1α promotes the occurrence and development of CRC by increasing the ability of tumor cells to adapt to hypoxia and low energy supply [26]. In the anoxic environment, the reduction of HIF-1 ubiquitination and degradation leads to accumulation of dimerized HIF-1α and HIF-1β, which transfers to the nucleus, and binds to potential hypoxia-responsive element in the CXCR4 promoter, accelerating the proliferation and metastasis of cancer cells [27]. HIF-1α is an important regulatory factor in the upstream of VEGF gene. A study showed that HIF-1α could not only combine with the hypoxia-responsive element of VEGF gene to promote its transcription but also increase the stability of VEGF mRNA [28]. VEGF is known as the most powerful, specific, and direct angiogenesis-inducing factor [29], while angiogenic phenotype is the prerequisite for a solid tumor to undergo malignant growth and successful metastasis [30]. Furthermore, HIF-1 could activate ERK and Akt pathways, upregulate the expression of VEGF, induce tumor angiogenesis, increase vascular permeability, and promote the metastasis of tumor cells by activating the expression of CXCR4 [31]. The results of the present study showed that CLG could significantly inhibit the increase in the protein levels of HIF-lα and VEGF in the transplanted tumor and the protein level of VEGF in serum, indicating the implication of inhibiting HIF-1α expression in the protection of CLG against the invasion and metastasis of CRC.

The degradation and destruction of extracellular matrix (ECM) and basement membrane (BM) of tumor cell surface play important roles in tumor metastasis [32,33], to which MMP-2 and MMP-9 are recognized as the critical contributors. In CRC, upon biding of SDF-1 to CXCR4, a number of G protein-coupled signaling pathways are activated, such as PI3K, Src Akt, MAPK, and NF-κB resulting in the increased expression of MMP-2 [34–37]. As the main component of BM, collagen IV is the substrate of MMP-2 and MMP-9 [38,39]. The results of the present study showed that CLG could significantly decrease the phosphorylation of PI3K and Akt and the protein level of MMP-2 in the transplanted tumor and reduce the degradation of collagen IV in the transplanted tumor, providing further insight for understanding the mechanisms thereby CLG inhibits the proliferation of CRC cells and liver metastasis.

Interestingly, despite of comparable effects being observed for high dose of CLG and Capecitabine on the inhibition of CRC metastasis and related signaling, the body weight of the nude mice in the Capecitabine group significantly decreased after chemotherapy, while that in high dose of CLG group was not affected implying a potential supremacy of CLG over Capecitabine in the clinical application for the patients with CRC.

The present study has some limitations. (1) CLG is a compound Chinese medicine containing multiple components. It is unclear what is the contribution of each component to the effects observed. (2) Although a number of signaling was found being affected by CLG that may account for the protective role of CLG on CRC metastasis, the exact target (s) for CLG to act remains unknown. (3) The effect of CLG on LoVo cells and its effect on SDF-1/CXCR4 expression were not verified *in vitro*. (4) The effect of CLG on the survival rate of CRC model in the nude mice needs to be studied by further investigation, which may help evaluate the feasibility of this drug for clinic use.

## Conclusions

In conclusion, the present study demonstrated the potential of CLG to inhibit liver metastasis of CRC in a nude mice model, which may be mediated by HIF-1α/SDF-1α-CXCR4/PI3K-Akt signaling pathway.

## Supporting information

S1 Table (DOCX)

S2 Table (DOCX)

S3 Table (DOCX)

S4 Table (DOCX)

S5 Table (DOC)

S6 Table (DOC)

S1 Fig (TIF)

S2 Fig (TIF)

S3 Fig (TIF)
