# Supplementary material for: Inhibitory effects of Chanling Gao on the proliferation and liver metastasis of transplanted colorectal cancer in nude mice
Source: PLoS One. 2019 Feb 21;14(2):e0201504. doi: 10.1371/journal.pone.0201504 (PMC6383928; doi:10.1371/journal.pone.0201504)
Supplement: S1 Table — (DOCX) [file pone.0201504.s001.docx]

**S1 Table. Body weight of colorectal cancer nude mouse model**

| **Date** | **n=6** | **Model(g)** | **Capecitabine(g)** | **CLGL(g)** | **CLGH(g)** |
| --- | --- | --- | --- | --- | --- |
| **2016-12-29** | **n1** | **16.40** | **17.10** | **19.80** | **18.40** |
|  | **n2** | **16.80** | **17.20** | **19.80** | **16.50** |
|  | **n3** | **16.10** | **15.50** | **16.70** | **17.40** |
|  | **n4** | **17.60** | **18.10** | **15.90** | **18.40** |
|  | **n5** | **18.40** | **17.00** | **15.60** | **17.60** |
|  | **n6** | **15.50** | **16.50** | **17.00** | **17.40** |
|  |  | **16.80±1.05** | **16.90±0.86** | **17.47±1.88** | **17.62±0.72** |
|  |  |  |  |  |  |
| **2016-12-31** | **n1** | **16.50** | **17.90** | **21.00** | **18.50** |
|  | **n2** | **17.90** | **16.50** | **20.20** | **16.50** |
|  | **n3** | **17.10** | **16.30** | **16.70** | **17.50** |
|  | **n4** | **17.40** | **18.20** | **15.90** | **18.40** |
|  | **n5** | **18.20** | **17.10** | **16.30** | **17.50** |
|  | **n6** | **15.20** | **17.00** | **17.30** | **17.10** |
|  |  | **17.42±0.67** | **17.20±0.84** | **18.02±2.39** | **17.68±0.81** |
|  |  |  |  |  |  |
| **2017-1-2** | **n1** | **16.70** | **17.10** | **20.50** | **19.30** |
|  | **n2** | **18.80** | **17.20** | **20.20** | **17.00** |
|  | **n3** | **16.40** | **16.10** | **16.70** | **17.90** |
|  | **n4** | **17.80** | **17.90** | **16.20** | **19.20** |
|  | **n5** | **18.10** | **17.80** | **16.80** | **18.00** |
|  | **n6** | **15.40** | **16.20** | **17.50** | **17.40** |
|  |  | **17.20±1.25** | **17.05±0.77** | **17.98±1.88** | **18.13±0.94** |
|  |  |  |  |  |  |
| **2017-1-4** | **n1** | **16.90** | **16.00** | **20.80** | **18.90** |
|  | **n2** | **19.20** | **17.30** | **20.50** | **17.50** |
|  | **n3** | **16.40** | **15.70** | **15.90** | **18.20** |
|  | **n4** | **18.70** | **18.30** | **16.90** | **19.90** |
|  | **n5** | **18.20** | **16.00** | **17.40** | **18.40** |
|  | **n6** | **16.60** | **16.70** | **18.10** | **18.30** |
|  |  | **17.67±1.19** | **16.67±0.99** | **18.27±1.98** | **18.53±0.81** |
|  |  |  |  |  |  |
| **2017-1-6** | **n1** | **17.30** | **15.90** | **19.90** | **19.10** |
|  | **n2** | **18.80** | **16.40** | **19.80** | **17.40** |
|  | **n3** | **16.60** | **15.20** | **15.80** | **18.60** |
|  | **n4** | **18.40** | **18.10** | **15.70** | **19.90** |
|  | **n5** | **18.20** | **15.50** | **18.00** | **18.30** |
|  | **n6** | **17.30** | **17.00** | **19.30** | **19.40** |
|  |  | **17.77±0.83** | **16.35±1.07** | **18.08±1.93** | **18.78±0.88** |
|  |  |  |  |  |  |
| **2017-1-8** | **n1** | **17.10** | **15.60** | **18.60** | **18.70** |
|  | **n2** | **19.00** | **16.40** | **19.90** | **17.50** |
|  | **n3** | **17.60** | **15.20** | **15.60** | **19.00** |
|  | **n4** | **17.10** | **17.90** | **15.60** | **19.10** |
|  | **n5** | **17.90** | **16.70** | **17.70** | **18.20** |
|  | **n6** | **16.80** | **16.80** | **18.60** | **18.80** |
|  |  | **17.58±0.80** | **16.43±0.96** | **17.67±1.75** | **18.55±0.60** |
|  |  |  |  |  |  |
| **2017-1-10** | **n1** | **16.90** | **16.00** | **19.80** | **19.10** |
|  | **n2** | **19.20** | **16.70** | **20.60** | **17.40** |
|  | **n3** | **18.00** | **14.50** | **15.60** | **18.70** |
|  | **n4** | **17.10** | **18.10** | **14.80** | **19.60** |
|  | **n5** | **18.00** | **16.60** | **17.70** | **18.50** |
|  | **n6** | **17.90** | **17.00** | **19.10** | **18.90** |
|  |  | **17.85±0.82** | **16.48±1.19** | **17.93±2.34** | **18.70±0.74** |
|  |  |  |  |  |  |
| **2017-1-12** | **n1** | **16.50** | **15.80** | **19.20** | **18.60** |
|  | **n2** | **18.30** | **15.30** | **20.30** | **16.60** |
|  | **n3** | **17.60** | **13.30** | **16.20** | **19.00** |
|  | **n4** | **16.90** | **16.90** | **15.70** | **18.90** |
|  | **n5** | **17.50** | **16.60** | **17.50** | **18.30** |
|  | **n6** | **16.90** | **16.40** | **18.30** | **18.10** |
|  |  | **17.28±0.65** | **15.72±1.32** | **17.87±1.76** | **18.25±0.88** |
|  |  |  |  |  |  |
| **2017-1-14** | **n1** | **16.50** | **15.90** | **18.90** | **18.60** |
|  | **n2** | **18.20** | **15.00** | **20.30** | **17.30** |
|  | **n3** | **17.40** | **12.60** | **15.60** | **19.40** |
|  | **n4** | **16.80** | **16.90** | **15.40** | **18.80** |
|  | **n5** | **17.80** | **17.20** | **17.10** | **18.30** |
|  | **n6** | **16.80** | **16.00** | **19.00** | **18.30** |
|  |  | **17.25±0.66** | **15.60±1.66** | **17.72±2.00** | **18.45±0.69** |
|  |  |  |  |  |  |
| **2017-1-16** | **n1** | **16.70** | **15.70** | **18.30** | **18.00** |
|  | **n2** | **18.20** | **15.10** | **20.30** | **17.10** |
|  | **n3** | **17.20** | **13.80** | **15.80** | **19.50** |
|  | **n4** | **16.60** | **16.80** | **15.70** | **18.40** |
|  | **n5** | **17.50** | **16.70** | **16.40** | **18.30** |
|  | **n6** | **16.50** | **16.70** | **19.10** | **17.90** |
|  |  | **17.12±0.66** | **15.80±1.19** | **17.60±1.91** | **18.20±0.78** |
|  |  |  |  |  |  |
|  |  |  |  |  |  |
| **2017-1-18** | **n1** | **17.20** | **14.60** | **17.70** | **18.00** |
|  | **n2** | **18.50** | **15.50** | **20.10** | **16.90** |
|  | **n3** | **17.70** | **13.10** | **15.40** | **19.20** |
|  | **n4** | **16.80** | **16.20** | **15.70** | **18.30** |
|  | **n5** | **17.00** | **16.70** | **16.40** | **18.80** |
|  | **n6** | **16.20** | **16.40** | **19.30** | **18.00** |
|  |  | **17.23±0.79** | **15.42±1.36** | **17.43±1.94** | **18.20±0.79** |
|  |  |  |  |  |  |
| **2017-1-20** | **n1** | **16.80** | **14.90** | **17.20** | **18.30** |
|  | **n2** | **18.30** | **16.10** | **20.10** | **17.70** |
|  | **n3** | **16.90** | **13.30** | **15.80** | **18.70** |
|  | **n4** | **16.20** | **16.00** | **15.90** | **18.80** |
|  | **n5** | **16.70** | **16.50** | **16.10** | **19.00** |
|  | **n6** | **15.50** | **16.70** | **19.40** | **18.30** |
|  |  | **16.73±0.93** | **15.58±1.28** | **17.42±1.89** | **18.47±0.47** |
