# Supplementary material for: Inhibitory effects of Chanling Gao on the proliferation and liver metastasis of transplanted colorectal cancer in nude mice
Source: PLoS One. 2019 Feb 21;14(2):e0201504. doi: 10.1371/journal.pone.0201504 (PMC6383928; doi:10.1371/journal.pone.0201504)
Supplement: S2 Table — (DOCX) [file pone.0201504.s002.docx]

**S2 Table . Inhibitory effect of CLG on the growth of the transplanted tumor in CRC nude mice (mean ± SEM, *n* = 6)**

| **Group** | **Tumor weight (g)** | **Inhibition rate of**  **Tumor weight** | **Tumor volume (mm^3^)** | **Inhibition**  **rate of**  **Tumor volume** |
| --- | --- | --- | --- | --- |
| Model | 0.444±0.041 | - | 500.36±40.26 | - |
| Capecitabine | 0.16±0.028* | 63.32% | 171.95±35.12* | 65.63% |
| CLGL | 0.29±0.025*^#^ | 35.26% | 318.87±38.08*^#^ | 36.27% |
| CLGH | 0.24+0.011* | 46.81% | 280.82±25.34* | 47.09% |

**S2 Table. Inhibitory effect of CLG on the growth of the transplanted tumor in CRC nude mice. CLGL: CLG low dose; CLGH: CLG high dose. *P＜0.05.vs Model; #P＜0.05.vs Capecitabine.**
