# Supplementary material for: Inhibitory effects of Chanling Gao on the proliferation and liver metastasis of transplanted colorectal cancer in nude mice
Source: PLoS One. 2019 Feb 21;14(2):e0201504. doi: 10.1371/journal.pone.0201504 (PMC6383928; doi:10.1371/journal.pone.0201504)
Supplement: S3 Table — (DOCX) [file pone.0201504.s003.docx]

**S3 Table. Liver metastasis in a nude mouse model of colorectal cancer（mean±SEM）**

| Group | n | Number of nude mice with liver metastases | Number of metastatic tumors  in the liver |
| --- | --- | --- | --- |
| Model | 6 | 6/6 | 15.17±1.376 |
| Capecitabine | 6 | 3/6 | 7.00±1.528* |
| CLGL | 6 | 5/6 | 12.80±1.463 |
| CLGH | 6 | 3/6 | 8.33±0.882* |

**S3 Table. The number of metastases of the transplanted tumor in the liver from different groups. Data are mean ± SEM (*n* = 6). **P*＜0.05 vs Model.**
