# Supplementary material for: Inhibitory effects of Chanling Gao on the proliferation and liver metastasis of transplanted colorectal cancer in nude mice
Source: PLoS One. 2019 Feb 21;14(2):e0201504. doi: 10.1371/journal.pone.0201504 (PMC6383928; doi:10.1371/journal.pone.0201504)
Supplement: S4 Table — (DOCX) [file pone.0201504.s004.docx]

**S4 Table. VEGF protein in nude mice transplanted tumor and serum（mean±SEM）**

| Group | n | VEGF in transplanted tumor | Serum VEGF |
| --- | --- | --- | --- |
| Model | 6 | 57.01±4.86 | 41.20±2.39 |
| Capecitabine | 6 | 30.23±5.74* | 23.78±3.00* |
| CLGL | 6 | 43.08±2.42 | 33.36±2.40 |
| CLGH | 6 | 35. 37 ±5.83* | 29.75±2.16* |

**S4 Table. Effects of CLG on the protein levels of VEGF in serum and the transplanted tumor tested by ELISA. Data are mean ± SEM (*n* = 6). **P*＜0.05 vs Model.**
