# Supplementary material for: Inhibitory effects of Chanling Gao on the proliferation and liver metastasis of transplanted colorectal cancer in nude mice
Source: PLoS One. 2019 Feb 21;14(2):e0201504. doi: 10.1371/journal.pone.0201504 (PMC6383928; doi:10.1371/journal.pone.0201504)
Supplement: S5 Table — (DOC) [file pone.0201504.s005.doc]

**S5 Table. MMP-2、Collgen IV protein in nude mice transplanted tumor（mean±SEM）**

| Group | n | MMP-2 | Collgen IV |
| --- | --- | --- | --- |
| Model | 6 | 27.54±3.48 | 6.34±1.80 |
| Capecitabine | 6 | 10.55±2.29* | 25.71±3.74* |
| CLGL | 6 | 23.13±2.08 | 12.58±1.67^#^ |
| CLGH | 6 | 11.85±4.97* | 20.97±2.23* |

**S5 Table. Effects of CLG on the protein levels of MMP-2 and collagen IV in the transplanted tumor tested by ELISA. Data are mean ± SEM (*n* = 6). **P*＜0.05 vs Model, *^#^P*＜0.05 vs Capecitabine.**
