# Supplementary material for: Inhibitory effects of Chanling Gao on the proliferation and liver metastasis of transplanted colorectal cancer in nude mice
Source: PLoS One. 2019 Feb 21;14(2):e0201504. doi: 10.1371/journal.pone.0201504 (PMC6383928; doi:10.1371/journal.pone.0201504)
Supplement: S6 Table — (DOC) [file pone.0201504.s006.doc]

**S6 Table. SDF-1α、CXCR4 protein in the liver of CRC nude mice（mean±SEM）**

| Group | n | SDF-1α | CXCR4 |
| --- | --- | --- | --- |
| Model | 6 | 119.00±7.43 | 30.47±4.91 |
| Capecitabine | 6 | 41.43±17.37* | 10.88±0.89* |
| CLGL | 6 | 90.17±13.06 | 20.78±1.85 |
| CLGH | 6 | 55.20±9.07* | 12.03±2.40* |

**S6 Table. The protein level of SDF-1α and CXCR4 in the liver of nude mice tested by ELISA. Data are mean ± SEM (*n* = 6). **P*＜0.05 vs Model.**
