# Supplementary figures and images for: Inhibitory effects of Chanling Gao on the proliferation and liver metastasis of transplanted colorectal cancer in nude mice
Source: PLoS One. 2019 Feb 21;14(2):e0201504. doi: 10.1371/journal.pone.0201504 (PMC6383928; doi:10.1371/journal.pone.0201504)

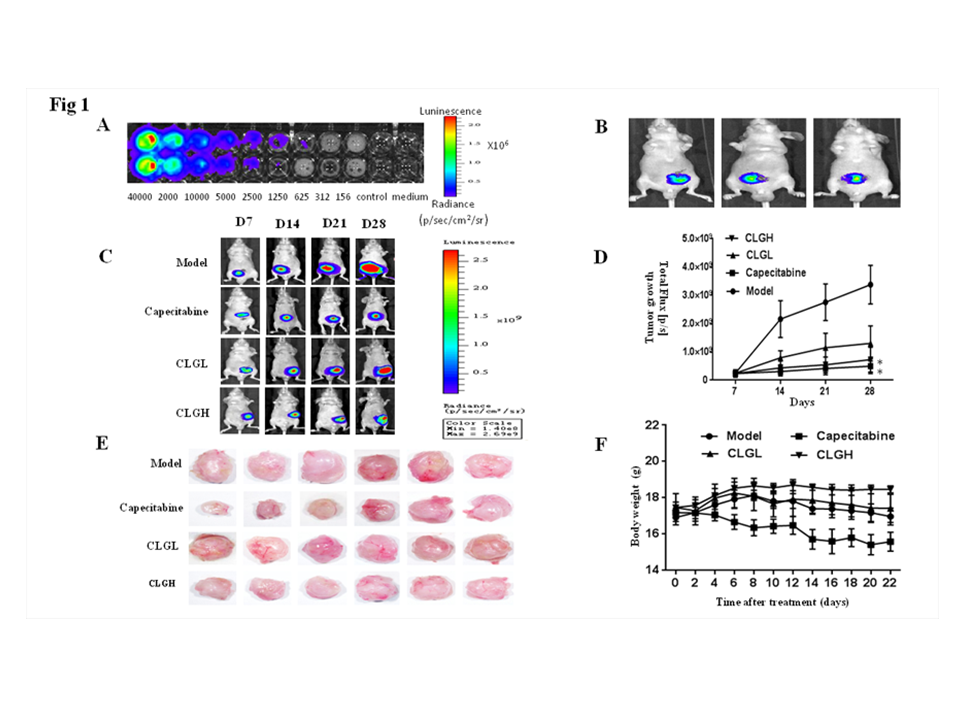

Supplement: S1 Fig — (TIF) [file pone.0201504.s007.tif]

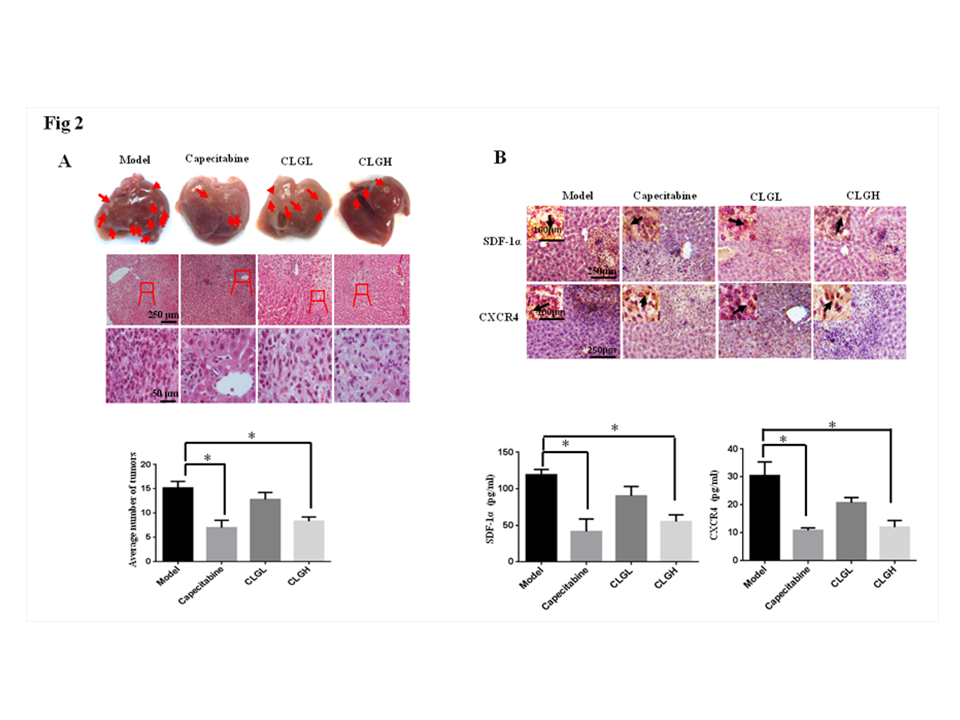

Supplement: S2 Fig — (TIF) [file pone.0201504.s008.tif]

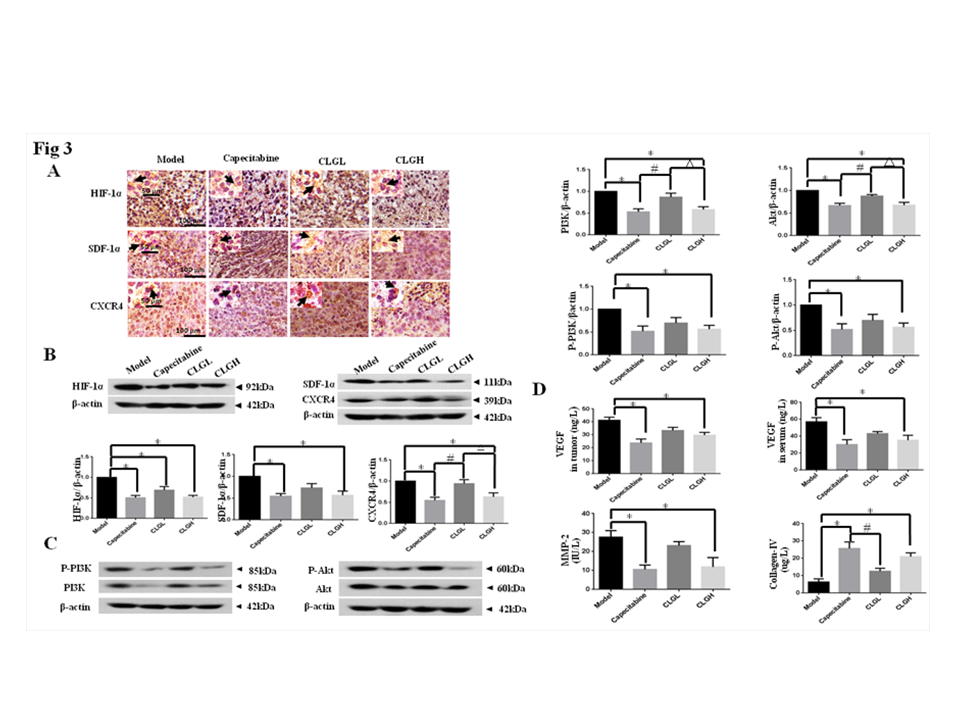

Supplement: S3 Fig — (TIF) [file pone.0201504.s009.tif]
